# Bridging the gap: unravelling plant centromeres in the telomere‐to‐telomere era

**DOI:** 10.1111/nph.20149

**Published:** 2024-09-27

**Authors:** Matthew Naish

**Affiliations:** ^1^ Department of Plant Sciences University of Cambridge Cambridge CB2 3EA UK

**Keywords:** centromere, CENH3, centromere evolution, centromere homeostasis, centromeric chromatin, satellite DNA, epigenetics

## Abstract

Centromeres are specific regions of the chromosomes that play a pivotal role in the segregation of chromosomes, by facilitating the loading of the kinetochore, which forms the link between the chromosomes to the spindle fibres during cell division. In plants and animals, these regions often form megabase‐scale loci of tandemly repeated DNA sequences, which have presented a challenge to genomic studies even in model species. The functional designation of centromeres is determined epigenetically by the incorporation of a centromere‐specific variant of histone H3. Recent developments in long‐read sequencing technology have allowed the assembly of these regions for the first time and have prompted a reassessment of fidelity of centromere function and the evolutionary dynamics of these regions.

**Table:** 

	Contents	
	Summary	2143
I.	Introduction	2143
II.	Architecture of centromeres	2144
III.	Evolution of satellite arrays	2144
IV.	CENH3 homeostasis	2145
V.	The epigenetic regulation of centromeres	2147
VI.	Conclusions and outlook	2148
	Acknowledgements	2148
	References	2148

## I. Introduction

When a cell divides, each daughter cell must receive an equal distribution of the genetic material. This depends on specialised regions called centromeres. Centromeres assemble a protein complex known as the kinetochore, which mediates the segregation by forming the vital connection between chromosomes and spindle microtubules.

In most organisms, the key determinant for the site of kinetochore assembly is epigenetic, requiring the deposition of a centromere‐specific variant of histone H3 called CENH3 in plants, CENP‐A in mammals, CID in flies, and Cse4/Cnp1 in yeast (Talbert & Henikoff, 2020).

The complex mechanisms governing centromere establishment, maintenance and degradation in many species have remained elusive, due to the highly repetitive structures typically associated with centromeric DNA. Historically these regions presented challenges to assembly, leaving significant gaps in genome assemblies. However, advances in long‐read sequencing technologies from Oxford Nanopore Technologies and Pacific Biosciences have allowed the complete assembly of these regions (Naish *et al*., 2021; Altemose *et al*., 2022).

Despite their conserved function, centromeres display considerable diversity and structural plasticity. Ranging in size from point centromeres in budding yeast (*c*. 125 bp), regional centromeres (35–110 kb) in fission yeast (Talbert & Henikoff, 2020) to megabase‐scale regions in *Arabidopsis thaliana* and humans, that are the site of multiple CENH3 nucleosomes and kinetochore complexes (Naish *et al*., 2021; Altemose *et al*., 2022).

The availability of telomere‐to‐telomere assemblies is changing our perspective of centromere biology. In this review I will discuss the insights provided into the main architectural types and evolution of plant centromeres, and discuss recent work investigating how the epigenetic environment, including patterns of CENH3 nucleosome occupancy, role of heterochromatin and DNA cytosine methylation, can influence fidelity of centromere function.

## II. Architecture of centromeres

**Fig. 1:**
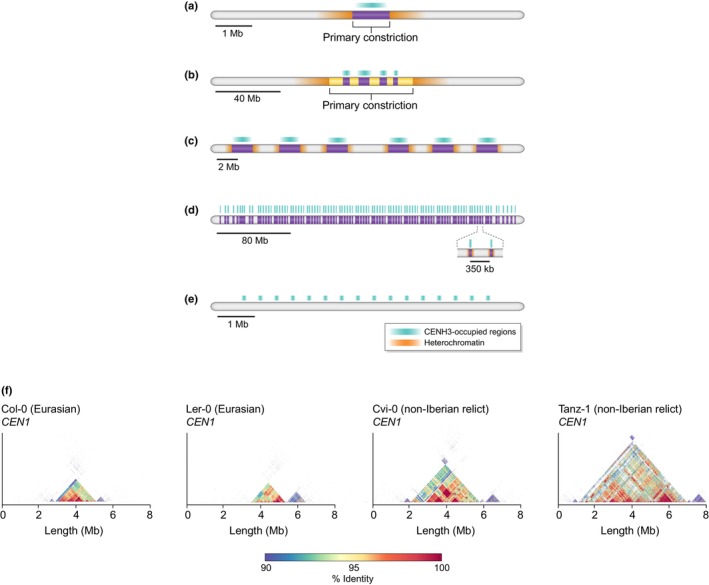
Schematic representing centromere architectures and example of intraspecies centromere diversity in Arabidopsis. (a) Monocentric architecture where there is a single centromere array (either transposon or satellite repeat) supports the loading of CENH3, such as found in *Arabidopsis thaliana* (Naish *et al*., 2021). (b) Metapolycentric architecture where multiple loci support the loading of CENH3 but act similarly to a monocentric chromosome during cell division, such as found in *Pisum sativa* (Macas *et al*., 2023). (c) Holocentric architecture where multiple evenly spaced arrays support the loading of CENH3 along the chromosome, such as found in *Chionographis japonica* (Naish & Henderson, 2024). (d) Holocentric architecture where thousands of small repeat arrays support the loading of CENH3 along the whole chromosome, such as found in *Rhynchospora pubera* (Hofstatter *et al*., 2022). (e) Nonrepeat‐based holocentric where kinetochores assemble without sequence specificity along the chromosomes in regions of low‐nucleosome turnover, such as found in *Caenorhabditis elegans* (Talbert & Henikoff, 2020). Centromere arrays (either transposon or satellite) highlighted in purple, the location of the CENH3‐occupied regions highlighted in green, and heterochromatin highlighted in orange. (f) StainedGlass identity heatmap of *CEN178* satellite arrays found on chromosome 1 of Col‐0, Ler‐0, Cvi‐0 and Tanz‐1 accessions (Wlodzimierz *et al*., 2023).

Centromeres perform a conserved function across eukaryotes, yet the associated DNA sequences are extremely variable in size, structure and organisation, both within and between species (Fig. 1; Henikoff *et al*., 2001). In plants, many species are monocentric, where a single chromosome region localises CENH3 often located on a satellite or transposon array (Fig. 1a). In satellite centromeres, repeat monomers typically range from 100 to 200 bp in length, supporting individual CENH3 nucleosomes. For example, in *Arabidopsis*, the centromeric repeat monomers (*CEN178*) are 178 bp long (Naish *et al*., 2021; Wlodzimierz *et al*., 2023).

However, alternative architectures in which kinetochores load at multiple sites distributed along individual chromosomes (holocentric) have evolved independently at least 16 times across eukaryotic lineages including in plant species such as *Rhynchospora* (Melters *et al*., 2012; Hofstatter *et al*., 2022; Fig. 1c–e). Additionally, meta‐polycentric species such as *Pisum sativum*, where multiple CENH3‐occupied satellites regions are separated by megabases of intervening sequences but act as a singular CENH3 focus during cell division and may represent an evolutionary transition between monocentric and holocentric architectures (Macas *et al*., 2023; Fig. 1b).

The functional role of repeats in plant centromeres is currently unclear. New genomes are revealing plant species, such as maize and potato, that contain chromosomes with centromeric satellite/transposon arrays and chromosomes that lack such arrays (Bao *et al*., 2022; Chen *et al*., 2023). This contrasts within humans and mice where the α‐satellite repeat monomer can contain a 17‐bp binding site, which recruits kinetochore protein CENP‐B, which directly facilitates CENP‐A incorporation (Masumoto *et al*., 1989).

An influential argument for functional evolution of centromeric arrays was proposed in the centromere drive model (Henikoff *et al*., 2001). In this model, asymmetric cell divisions, such as those in female meiosis (where only one of the four meiotic products is transmitted to the next generation), create selective pressure for centromeric variants that bias their orientation during the first meiotic division, ensuring their inheritance.

Consistent with this model, a high level of repeat sequence variation has been observed within plant genomes. For example, in *Arabidopsis*, numerous *CEN178* satellite polymorphisms exist, many of which are private to individual chromosomes. Within these arrays, there are signatures of sequence homogenisation, with CENH3 enrichment occurring across the most homogeneous monomers (Maheshwari *et al*., 2017; Naish *et al*., 2021; Wlodzimierz *et al*., 2023), with a similar pattern observed in rice (Song *et al*., 2021).

The most direct evidence for this model is observed in monkeyflowers, in which a large duplication of the satellite sequence results in transmission to 90% of offspring through female meiosis (Finseth *et al*., 2021).

## III. Evolution of satellite arrays

The mechanism behind the formation of these repeat arrays is unclear. An early model was ‘unequal crossover’ which posited double‐strand breaks could be repaired using nonallelic locations, leading to unequal out‐of‐register exchange, generating duplications and deletions of the intervening repeats (Smith, 1976). However, analysis of 66 *Arabidopsis* accessions reveals the pericentromeric sequences flanking the repeat arrays are in linkage, despite internal satellite changes (Wlodzimierz *et al*., 2023). These results are consistent with the centromeric haplotypes reported in humans (Altemose *et al*., 2022) and suggest rearrangements through unequal meiotic crossover is unlikely as a general mechanism for centromere evolution and that smaller scale gene conversions or inter‐sister crossover pathways are more likely.

Recent work has suggested break‐induced replication (BIR) as a mechanism of generating the observed variation. In this model, the persistent core of the kinetochore presents a barrier to replication, which results in the replication fork collapsing, creating a double‐strand break. Re‐initiation occurs through the BIR pathway, which is likely to be out‐of‐register within a tandem array, generating duplications or deletions within the array (Showman *et al*., 2023). This may not generate satellites directly, as human neocentromeres do not show any sequence changes at the new centromere location after 200 generations (Murillo‐Pineda *et al*., 2021). However, Showman *et al*. quantified the copy number of a Chromosome 11 satellite repeat over 20 generations, revealing significant expansion and contractions in satellite abundance (7–100%) dependent on RAD52 and PIF1, consistent with BIR. Suggesting once satellites become established, these pathways can rapidly turnover sequences through mitotic divisions.

In the holocentric species *Rhynchospora* (Cyperaceae), kinetochore attachments occur on relatively short (15–25 kb) arrays of Tyba monomers (172 bp) distributed at *c*. 350 kb intervals along the chromosome. In this system, Hofstatter *et al*. (2022) propose a role for TCR1 and TCR2 nonautonomous helitrons in the spread of the *Tyba* repeat arrays along the chromosome. This proposal is consistent with data from other species where transposable elements invade satellite repeat sequences, potentially seeding repeats or becoming the centromere associated sequences themselves (Talbert & Henikoff, 2020).

The contrast between repeat homogenisation and pericentromeric haplotype emergence is an unexplained feature of centromeres. As further complete genomes accumulate, it will be possible to analyse pan‐centromeric diversity within populations to reveal the mechanisms of centromere evolution.

## IV. CENH3 homeostasis

To understand plant centromeres further, questions remain regarding the mechanisms by which their locations are established, maintained, and degraded. The roles histone chaperons in the establishment of CENP‐A have been well‐characterised in animals and yeasts (Takeuchi *et al*., 2024). However, in plants, the role of DNA repeats and molecular mechanisms involved in deposition of CENH3 are still unclear.

In contrast to the relatively conserved canonical histone H3 variants, CENH3 proteins are highly variable between species, although diverged variants from *Lepidium oleraceum* and *Zea mays* can still functionally complement *cenh3* in *Arabidopsis* (Maheshwari *et al*., 2017). Across other species, the CENH3 proteins are deposited by distantly related or nonconserved histone chaperones, including HJURP in mammals, Scm3 in yeasts, and CAL1 in flies (Talbert & Henikoff, 2020).

**Fig. 2:**
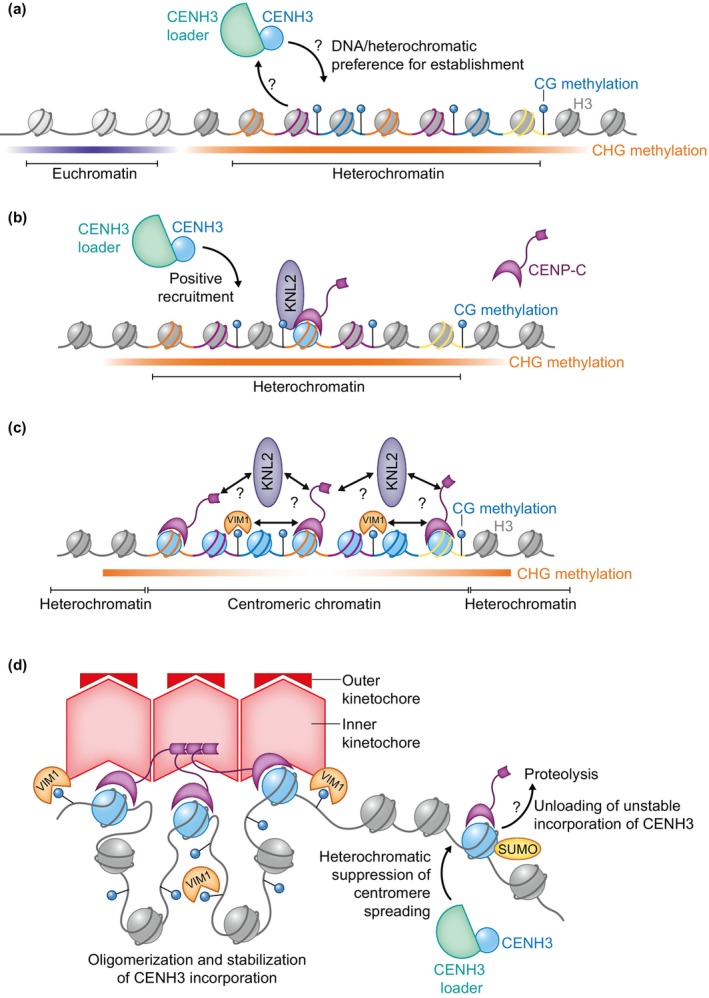
Model for CENH3 loading and centromere homeostasis in Arabidopsis. (a) CENH3 (green circle) is loaded into a heterochromatin region with high‐DNA methylation (H3; grey circles) within a CEN178 satellite repeat array. Loading is mediated by a histone chaperone, for example, NASP^SIM3^ (Le Goff *et al*., 2020; Takeuchi *et al*., 2024). The individual repeats are indicated by varying colours. (b) Once CENH3 has been loaded, it assembles inner kinetochore proteins, including CENP‐C and KNL2 (purple) (Ogura *et al*., 2004; Le Goff *et al*., 2020). Interactions between the CENH3 loader complex and kinetochore factors may create feed‐forward recruitment of CENH3. (c) The density of CENH3‐containing nucleosomes increases and reaches an equilibrium, the region undergoes a maturation establishing distinct centromeric chromatin, CENH3 incorporation is stabilised by oligomerisation of inner kinetochore proteins, for example, CENP‐C or KNL2 (Hara *et al*., 2023; Sissoko *et al*., 2024). VIM1 (orange) is known to bind and maintain methylation at CG sites and contributes to centromere fidelity, potentially through the compaction of the centromeric region allowing oligomerisation to occur (potential interactions represented by arrows). (d) Stable incorporation of CENH3 nucleosomes allow recruitment of inner and outer kinetochore complexes (red). At the boundaries of the centromeric region, the heterochromatin or CENH3 SUMOylation (yellow circle) may supress the establishment of new CENH3 regions suppressing the formation dicentric chromosomes. Adapted from Naish & Henderson (2024).

In plants, no CENH3‐specific chaperones have been identified. However, a homolog of the chaperone nuclear autoantigenic sperm protein (NASP) found in animals and yeast was characterised as a H3 chaperone in *Arabidopsis* (NASP^sim3^) (Maksimov *et al*., 2016). This chaperone was reported to bind CENH3 *in vitro*, with knockdown lines indicating a *c*. 30% reduction CENH3 signal at the centromere (Le Goff *et al*., 2020). It has recently been implicated in the deposition of CENH3 after fertilisation (Takeuchi *et al*., 2024), potentially providing an important piece of this pathway (Fig. 2a).

During evolution, arrays may be split through chromosome rearrangements, or novel arrays can emerge leading to multiple distinct satellite regions on the chromosome. This requires regulation of ectopic loading of CENH3 to avoid the chromosomal instability of dicentric chromosomes. This mechanism is better understood in humans where a positive feedback‐loop reinforces CENP‐A deposition at a given locus, mediated through HJURP directly binding to kinetochore protein Mis18 and CENP‐A:H4 tetramers (Barnhart *et al*., 2011). It remains to be seen whether a homeostatic mechanism exists to regulate centromere size (Fig. 1b,c). However, in *Arabidopsis* there is a large range of centromeric array sizes observed (*c*. 1.5–6.5 Mb) between accessions (Fig. 1f) but the region of CENH3 enrichment remains similar (1–2 Mb) (Wlodzimierz *et al*., 2023). This contrasts with the much smaller 100–200 kb regions of CENP‐A enrichment in humans, potentially indicating a stronger homeostatic limit in human cells despite having the larger genome (Altemose *et al*., 2022; Logsdon *et al*., 2024).

As increasing assemblies are characterised the intraspecies variation in CENH3 regions is becoming apparent. The location of the core CENH3 region is reported to vary between the lines in soybean, wheat and maize (Hufford *et al*., 2021; Liu *et al*., 2023; Zhao *et al*., 2023). One plausible explanation for this could be the increased selection placed on crop genomes through domestication, resulting in the fixation of new centromere locations. This also seems to be the case in mammals, where assembly of a second cell line (CHM1) revealed a quarter of the human centromeres differed in kinetochore position by > 500 kb compared to the CHM13 (Logsdon *et al*., 2024).

The emergence of evolutionary new centromeres without large‐scale chromosome rearrangements is reported to play a role in reproductive isolation and speciation in plants (Mandáková *et al*., 2020). The mechanism of centromere migration remains elusive, requiring the seeding of a new functional centromere, and simultaneous decay of the old centromere to avoid dicentric‐chromosome instability or fragmentation. There is some evidence for this, as wheat CENH3 preferentially associates with rye repeat satellites following chromosome fusions, despite the chromosome retaining the native wheat repeats (Karimi‐Ashtiyani *et al*., 2021). However, the seeding of new centromeres does not directly result in the inactivation of established loci. In maize, deposition of CENH3 via LexA fusions leads to sustained establishment of ectopic CENH3 but results in dicentric chromosomes, chromosome instability, and formation of autonomously replicating mini‐chromosomes (Dawe *et al*., 2023).

Mérai *et al*. (2014), demonstrated that the chaperone CDC48A directs complete centromere disassembly by actively unloading SUMOylated CENH3 from native centromeres in pollen vegetative nuclei. Yet, it is unclear whether this mechanism more broadly regulates CENH3 distribution within centromeres (Fig. 2d).

These studies highlight the dynamic regulation of CENH3, including its potential for rapid migration to new DNA sequences. Investigating the factors and developmental stages that influence the rate and extent of centromere migration remains an important area for further research.

## V. The epigenetic regulation of centromeres

Centromeres are often located in regions of heterochromatin but contain a distinct chromatin state (Naish *et al*., 2021). How the wider chromatin environment contributes to the establishment, maintenance or fidelity of the centromeric region remains an open question.

In *Arabidopsis*, heterochromatin is enriched in features such as H3K9me2, but analysis of mutants impaired in the maintenance of these modifications have not reported defects on chromosome segregation (Yelina *et al*., 2012). Work in fission yeast, has shown H3K9me2 is necessary for *de novo* assembly of CENP‐A, but not fidelity of centromere function (Folco *et al*., 2008). However, inactivation of one of the centromeric regions in fission yeast undergoing experimental dicentric formation required increased and ongoing H3K9me2 accumulation and loss of histone acetylation over the central core region (Sato *et al*., 2012). These results indicate the potential for distinct roles for H3K9me2 or wider heterochromatic features between establishment and regulation of centromeric regions (Fig. 2).

A widespread feature of heterochromatin is DNA methylation. Studies across eukaryotes, including plants and humans, have shown that centromeres are often DNA methylated. However, the context of the methylation can differ. For example, in *Arabidopsis*, the CENH3 enriched regions are characterised by high levels of CG methylation, but reduced CHG methylation compared to the neighbouring heterochromatin (Naish *et al*., 2021; Wlodzimierz *et al*., 2023). This is likely to be due to the displacement of canonical H3 modified by H3K9me2 and breaking the maintenance loop of CHG methylation. This contrasts with humans where the centromeres are hypomethylated for CG methylation at the site of CENP‐A deposition (Altemose *et al*., 2022).

In *Arabidopsis*, Marimuthu *et al*., reported a functional link between DNA methylation and centromere fidelity. In this study, mutants in VIM1 (ortholog of mammalian UHRF1) which encodes a conserved E3‐ligase required for maintenance of CG methylation, increased the frequency of haploid induction by CENH3‐tailswap‐GFP lines (Marimuthu *et al*., 2021). This implies that the epigenetic change caused by *vim1* increased the functional mismatch between centromeres, leading to greater chromosome mis‐segregation and rates of genome elimination (Marimuthu *et al*., 2021).

Recent structural work is also raising questions about the role of inner kinetochore proteins in regulating functional structure of centromeric chromatin (Pesenti *et al*., 2022; Yatskevich *et al*., 2022). *In vivo* studies that show dimers of CENP‐C stably bind and link two nucleosomes, with oligomerisation of CENP‐C and CENP‐T facilitating further assembly of all kinetochore subunits similar those of endogenous human kinetochores (Hara *et al*., 2023; Sissoko *et al*., 2024). Thus, the accumulation of inner kinetochore factors may regulate higher‐order structure of high‐density CENH3 regions.

CENP‐C is conserved in plants (Ogura *et al*., 2004), interacting with a centromere licensing factor KNL2 (Kinetochore Null2) (Lermontova *et al*., 2013) so similar oligomerisation may operate during plant kinetochore formation. Although this may still be dependent on other features such as DNA methylation as cytological analysis of *vim1* show decondensed satellite arrays with reduced CENH3 signal (Woo *et al*., 2007; Fig. 2c).

In the holocentric species *Rhynchospora pubera*, the epigenetic pattern of the centromere units mirrors that of monocentric centromeres despite the extensive genome reorganisation. This indicates that the epigenetic control of repeat‐based centromeres is evolutionarily conserved in both monocentric and holocentric organisms (Hofstatter *et al*., 2022).

Together, these results indicate a complex relationship between CENH3 deposition and the wider chromatin environment (Fig. 2). In plants, analysis of epigenetic mutants using the newly assembled centromere sequences could provide the key to untangling this regulation and understanding how they impact centromere fidelity.

## VI. Conclusions and outlook

The field of centromere research is being transformed by telomere‐to‐telomere assemblies, which offer unprecedented insights into these previously enigmatic genome regions. To characterise the dynamic chromatin features and higher‐order structures of centromeres and other repeats, innovative experimental approaches will be essential.

New assemblies from projects like the ‘Darwin Tree of Life’, allow the examination of intra and interspecific centromere diversity, which will be integral to enhance our understanding of the evolutionary dynamics of repeat regions and associated chromatin.

The mechanisms by which centromeric sequences and chromatin come together to functionally determine the relative fidelity of the centromere regions remains an open question, with implications for both short and long‐term evolution, as well as wider population genetics.

Moving forward, understanding the regulatory mechanisms of these regions will be crucial to develop new tools for crop development such as haploid induction technologies, and pioneering innovations for engineering biology, including artificial chromosomes.

## Competing interests

None declared.
